# Combined use of irinotecan with histone deacetylase inhibitor belinostat could cause severe toxicity by inhibiting SN-38 glucuronidation *via* UGT1A1

**DOI:** 10.18632/oncotarget.15017

**Published:** 2017-02-02

**Authors:** Lingzhi Wang, Chong En Linus Chan, Andrea Li-Ann Wong, Fang Cheng Wong, Siew Woon Lim, Arunachalam Chinnathambi, Sulaiman Ali Alharbi, Lawrence Soon-U Lee, Ross Soo, Wei Peng Yong, Soo Chin Lee, Paul Chi-Lui Ho, Gautam Sethi, Boon Cher Goh

**Affiliations:** ^1^ Cancer Science Institute of Singapore, National University of Singapore, Singapore; ^2^ Department of Pharmacology, National University Health System, Singapore; ^3^ Department of Pharmacy, National University of Singapore, Singapore; ^4^ Department of Haematology-Oncology, National University Health System, Singapore; ^5^ Department of Botany and Microbiology, College of Science, King Saud University, Riyadh, Kingdom of Saudi Arabia; ^6^ School of Biomedical Sciences, Curtin Health Innovation Research Institute, Curtin University, Perth WA, Australia

**Keywords:** drug-drug interactions, irinotecan, SN-38, belinostat, UGT1A1

## Abstract

SN-38, the active metabolite of irinotecan, and histone deacetylase inhibitors (HDACis) such as belinostat, vorinostat and panobinostat, have all been shown to be deactivated by glucuronidation via UGTs. Since they all compete for UGTs for deactivation, we aimed to investigate the inhibitory effect of various HDACis on the glucuronidation of SN-38. This inhibitory effect was determined by measuring the formation rate of SN-38 glucuronide after SN-38 incubation with human recombinant UGT1A isoforms (1A1, 1A6, 1A7 and 1A9) and pooled human liver microsomes (HLM, wild type, UGT1A1*1*28 and UGT1A1*28*28 allelic variants), with and without HDACis. The data showed that belinostat at 100 and 200 µmol/L inhibited SN-38 glucuronidation via UGT1A1 in a dose-dependent manner, causing significant decrease in V_max_ and CL_int_ (*p* < 0.05) from 12.60 to 1.95 pmol/min/mg and 21.59 to 4.20 μL/min/mg protein respectively. Similarly, in HLMs, V_max_ dropped from 41.13 to 10.54, 24.96 to 3.77 and 6.23 to 3.30 pmol/min/mg, and CL_int_ reduced from 81.25 to 26.11, 29.22 to 6.10 and 5.40 to 1.34 µL/min/mg protein for the respective wild type, heterozygous and homozygous variants. Interestingly, belinostat at 200 µmol/L that is roughly equivalent to the average Cmax, 183 µmol/L of belinostat at a dose of 1,400 mg/m^2^ given intravenously once per day on days 1 to 5 every 3 weeks, was able to inhibit both heterozygous and homozygous variants to same extents (~64%). This highlights the potential clinical significance, as a large proportion of patients could be at risk of developing severe toxicity if irinotecan is co-administered with belinostat.

## INTRODUCTION

Combination therapy is often used for cancer patients in current clinical practice, as it has been shown to be more efficacious than monotherapy in combating cancer [1]. However, drug-drug interactions (DDIs) are an ongoing concern due to the narrow therapeutic windows of most chemotherapeutic agents and their potential for severe toxicity; irinotecan is one such agent whose interactions with other drugs may lead to severe toxicities such as diarrhoea and neutropenia [2, 3].

Irinotecan is an effective antitumour agent, displaying a broad range of clinical activities against neoplastic disorders such as colorectal, gynaecological and lung cancers [4, 5]. This drug is predominantly converted by carboxylesterases (CES) in the liver to SN-38, an active metabolite that is 100-1000 times more potent than irinotecan [6–9]. The potent SN-38 is then primarily deactivated by glucuronidation catalyzed by UGT1A1 to SN-38 glucuronide (SN-38G) [10–12]. Other UGT1A isoforms such as UGT1A6, UGT1A7 and UGT1A9, have also been reported to play a role in the formation of SN-38G [2, 13–15].

An illuminating example of toxicity caused by accumulation of SN-38 can be attributed to the functional polymorphism of UGT1A1. Genetic polymorphisms in UGT1A1 have been demonstrated to have great impact on SN-38 toxicity through influencing enzyme activity, leading to inter-individual differences in drug response [13, 16–18]. Notably, when compared to wildtype genotype, patients expressing the UGT1A1*28 allele experienced significant irinotecan treatment-related toxicity (*p* < 0.001) due to decreased (1.3 to 3.9 fold lower) enzymatic activity in liver microsomes. This resulted in reduced SN-38G formation and higher levels of SN-38 [3]. Similarly, any potential DDI with irinotecan that increases the serum levels of SN-38 could also cause severe undesirable toxicity. For instance, co-administration of ketoconazole and irinotecan to cancer patients resulted in a significant increase in the formation of SN-38, at least partially due to the inhibitory effect of ketoconazole on UGT1A1 [19,20]. Thus, it is important to investigate the probable interaction of irinotecan with other anti-cancer agents and to prevent such treatment-related toxicity.

Hydroxamic acid histone deacetylase inhibitors have emerged as a promising class of anti-cancer drugs in recent years [21,22]. They enhance histone acetylation and increase the expression of tumour suppressor genes, thereby inducing growth arrest and apoptosis of cancer cells [23–25]. It is also believed that they are capable of sensitising drug resistant cancer cells to anticancer drugs in combination therapy [26, 27]. The most commonly used HDACis in the hydroxamate class include belinostat, vorinostat and panobinostat. It has been shown that all 3 HDACis undergo intensive metabolism via phase II glucuronidation [28–30]. In particular, belinostat, like irinotecan, has recently been discovered to utilise the same phase II metabolic pathway involving the highly polymorphic enzyme, UGT1A1 [28]. Therefore, we hypothesise that HDACis may inhibit the UGT1A isoforms in phase II metabolism, resulting in reduced conversion of SN-38 to SN-38G and greater accumulation of SN-38.

Our objective was to investigate the potential presence of glucuronidation-mediated DDI in combination therapies of irinotecan with HDACis. Belinostat was approved for peripheral T-cell lymphoma (PTCL) by FDA recently [31], whereas vorinostat is FDA-approved for cutaneous T-cell lymphoma (CTCL) [32]. This investigation would then play a vital role in helping clinicians make more informed decisions regarding possible combination chemotherapy. Moreover, our results would also provide vital information to formulation scientists if these combinations of drugs are considered to be formulated together.

In this study, we assessed the inhibitory effects of belinostat on SN-38 glucuronidation *in vitro* using UGT1A1 enzymes, and its inhibitory effects were further confirmed using 3 types of pooled human liver microsomes (HLMs) (50 donor pool, UGT1A1*1*28 and UGT1A1*28*28 allelic variants). Significant associations were observed between the UGT1A1*28 polymorphisms and SN-38G formation rates in the absence and presence of belinostat. Besides, we also studied the possible inhibitory effects of other HDACis, including vorinostat and panobinostat on SN-38 glucuronidation.

## RESULTS

### LC-MS/MS method validation

**Figure 1:**
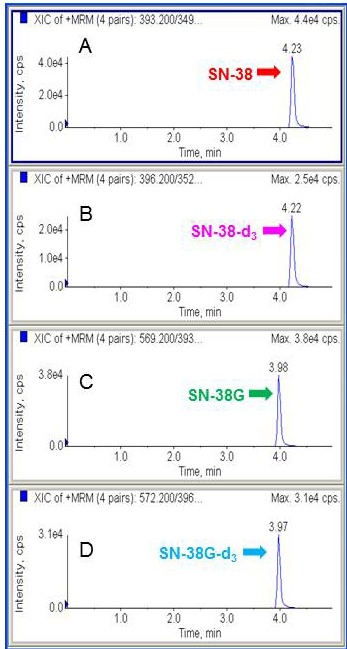
Representative chromatograms of SN-38, SN-38-d3, SN-38G and SN-38G-d3 Peak of SN-38 **A**. Peak of SN-38 internal standard using isotopic sister of the analyte, SN-38-d3 **B**. Peak of SN-38G **C**. Peak of SN-38G internal standard, SN-38G-d3 **D**.

The chromatographic data were acquired and analysed using the Analyst v1.4.2 software package (Applied Biosystems/MDS SCIEX). The LC-MS/MS analysis was highly specific and selective as the peaks have a symmetrical resolution, with no interference around the retention time (*t*_R_) of the analytes. The retention times were as follows: SN38 and SN-38-d3 eluting at *t*_R_ = 4.24 min, and SN-38G and SN-38G-d3 eluting at *t*_R_ = 3.97 min (Figure 1). Accuracy was expressed as the percentage of the mean value measured over the true value of QC samples, whereas precision at each QC concentration was expressed as the coefficient of variation by calculating the standard deviation as a percentage of the mean calculated concentration. Briefly, excellent linearity of SN-38 and SN-38G was demonstrated to be within the range of 10 to 500 nmol/L. A weighting factor of 1/x was used to minimise deviations from the slope of the curve at lower concentrations resulting from bigger variance at higher concentrations. Good linearity was achieved, with a coefficient of determination (r^2^) of > 0.996 for both SN-38 and SN-38G. This implied a strong linear relationship between the peak area ratio and concentration. The intra-day and inter-day precisions and accuracies for QC samples were summarised in Table 1. The intra-day precision was between 1.26-2.41% for SN-38 and 1.62-6.24% for SN-38G and the inter-day precision was 1.67-3.38% for SN-38 and 4.22-8.30% for SN-38G. The accuracy was between 91.14%-100.78% for SN-38 and 91.45%-105.60% for SN-38G. All these data met the requirement of the FDA guidelines [33]. Therefore, this method was considered sufficiently accurate, sensitive and reproducible for the simultaneous determination of SN-38 and SN-38G throughout a wide dynamic range.

**Table 1:** Intra-day and inter-day precision and accuracy of SN-38 (A) and SN-38G (B)

(A)	QC samples					
	Intra-day (*n* = 3)			Inter-day (*n* = 3)		
Nominal conc. (nmol/L)	Quantified conc. (Mean ± S.D, nmol/L)	Accuracy (%)	CV (%)	Quantified conc. (Mean ± S.D, nmol/L)	Accuracy (%)	CV (%)
30	27.33 ± 0.44	91.14	1.59	29.15 ± 1.80	97.28	6.24
150	147.00 ± 3.54	97.94	2.41	144.50 ± 2.33	96.20	1.62
350	352.67 ±4.46	100.78	1.26	346.00 ± 9.26	98.80	2.69

(B)	QC samples					
	Intra-day (*n* = 3)			Inter-day (*n* = 3)		
Nominal conc. (nmol/L)	Quantified conc. (Mean ± S.D, nmol/L)	Accuracy (%)	CV (%)	Quantified conc. (Mean ± S.D, nmol/L)	Accuracy (%)	CV (%)
30	27.43 ± 0.46	91.45	1.67	27.93 ± 1.93	93.18	6.92
150	143.67 ± 4.02	95.71	2.80	145.00 ± 6.10	96.60	4.22
350	369.00 ± 12.46	105.36	3.38	369.70 ± 30.68	105.60	8.30

### Effects of belinostat on SN-38 glucuronidation by UGT1A1

**Figure 2:**
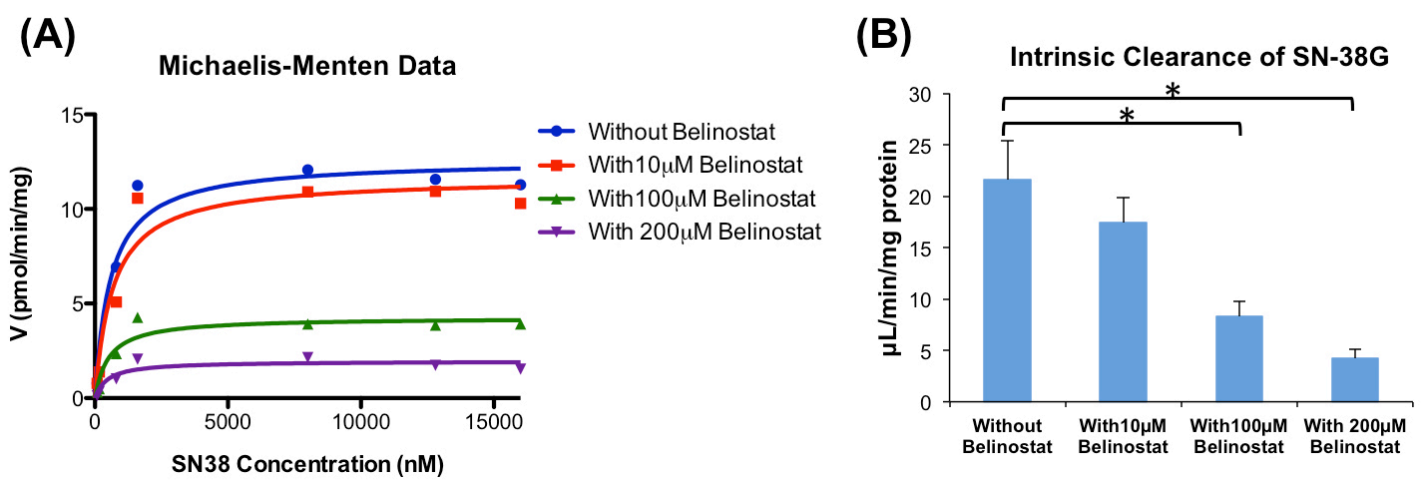
Enzyme kinetics of glucuronidation of SN-38 by UGT1A1 Michaelis-Menten graph of SN-38G formation when different concentrations of SN-38 were incubated with belinostat (0, 10, 100 and 200 μmol/L) **A**. Apparent intrinsic clearance of SN-38G **B**. By using a paired t-test, the incubations with 100 and 200 μmol/L belinostat were significantly different (*p* < 0.05) from the incubation without belinostat (*).

As the concentration of belinostat increased, the formation rate of SN-38G decreased in a dose-dependent manner, demonstrating an increase in the inhibition of SN-38 glucuronidation (Figure 2A), thus suggesting that the inhibition type was non-competitive. Upon addition of belinostat at 100 and 200 μmol/L, intrinsic clearance (CLint) was markedly decreased by ~ 62% (from 21.59 to 8.28 μL/min/mg protein) and 81% (from 21.59 to 4.20 μL/min/mg protein) respectively (*p* < 0.05) (Figure 2B).

### Effects of belinostat on SN-38 glucuronidation by pooled HLMs

**Figure 3:**
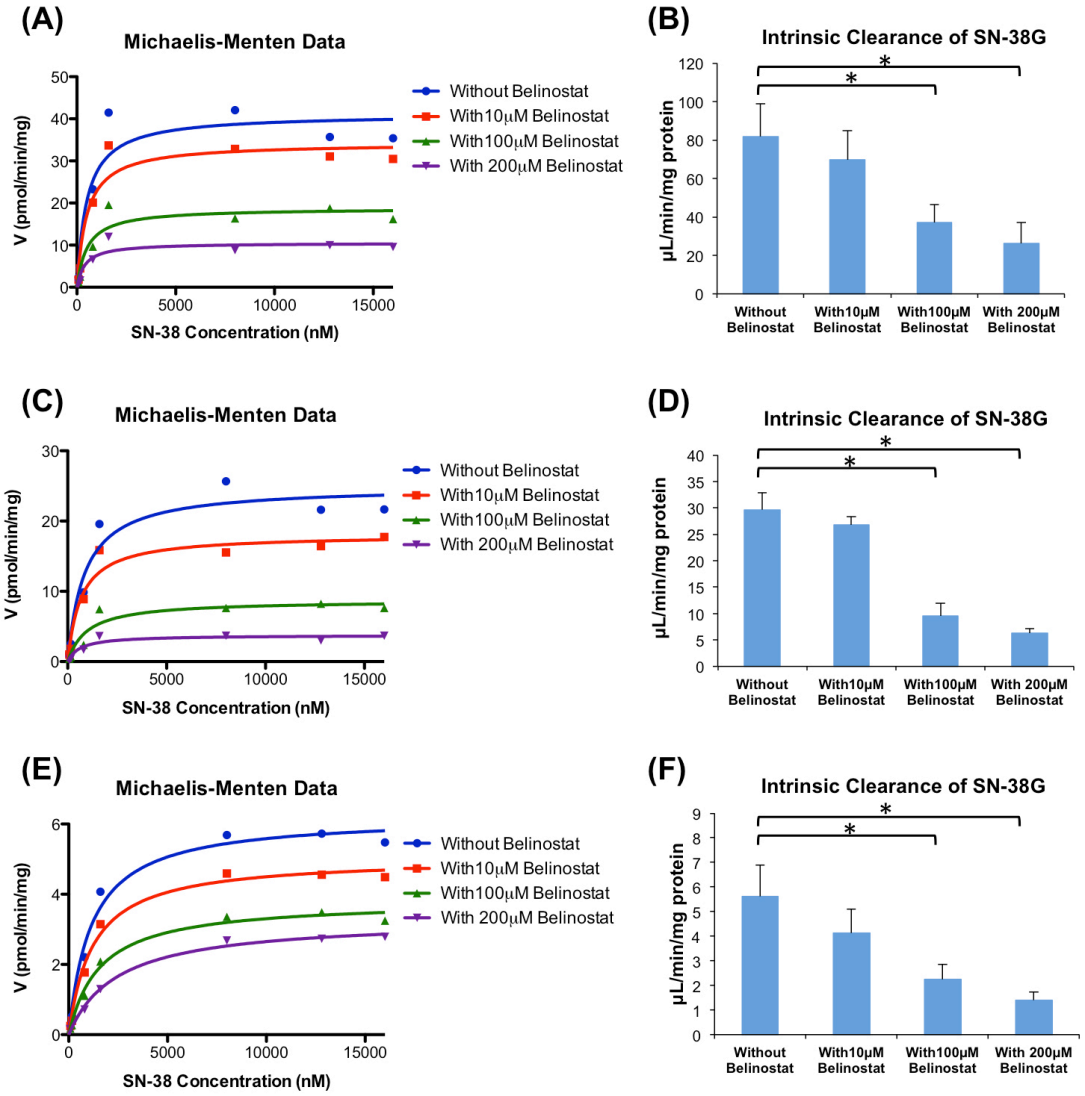
Enzyme kinetics of glucuronidation of SN-38 by human liver microsomes (HLM) Michaelis-Menten graphs of SN-38G formation when different concentrations of SN-38 were incubated with belinostat (0, 10, 100 and 200 μmol/L) and 6/6 genotype **A**., 6/7 genotype **C**. and 7/7 genotype **E**.; Apparent intrinsic clearance of SN-38G in 6/6 genotype **B**., 6/7 genotype **D**. and 7/7 genotype **F**. By using a paired t-test, the incubations with 100 and 200 μmol/L belinostat were significantly different (*p* < 0.05) from the incubation without belinostat (*).

Belinostat was also found to strongly inhibit SN-38 glucuronidation by the 3 types of pooled HLMs (50 donor pool, UGT1A1*1*28 and UGT1A1*28*28 allelic variants) in a dose-dependent manner (Figure 3). The 50 donor pool HLMs, were used to represent the wild-type homozygote for (TA)6TAA allele (6/6), while the UGT1A1*1*28 and UGT1A1*28*28 allelic variant HLMs are heterozygote (6/7) and homozygote (7/7) for (TA)7TAA allele, respectively. Based on the analysis of the Michaelis-Menten graphs (Figure 3A, 3C and 3E), belinostat also showed non-competitive inhibition against SN-38 glucuronidation by HLMs. In the 50 donor pool HLMs (6/6) (Figure 3B), belinostat showed the highest inhibitory effect at 200 μmol/L, significantly decreasing the CLint by 68%, from 81.25 to 26.11 μL/min/mg protein (*p* < 0.05). At 100 μmol/L of belinostat, CLint was also significantly decreased by 56%, from 81.25 to 36.01 μL/min/mg protein (*p* < 0.05). In the UGT1A1*1*28 (6/7) and UGT1A1*28*28 (7/7) allelic variant HLMs (Figure 3D and 3F), similar significant decreases were detected, where CLint dropped by 79% (from 29.22 to 6.10 µL/min/mg protein) and 75% (from 5.40 to 1.34 µL/min/mg protein), respectively at 200 μmol/L of belinostat (*p* < 0.05).

**Figure 4:**
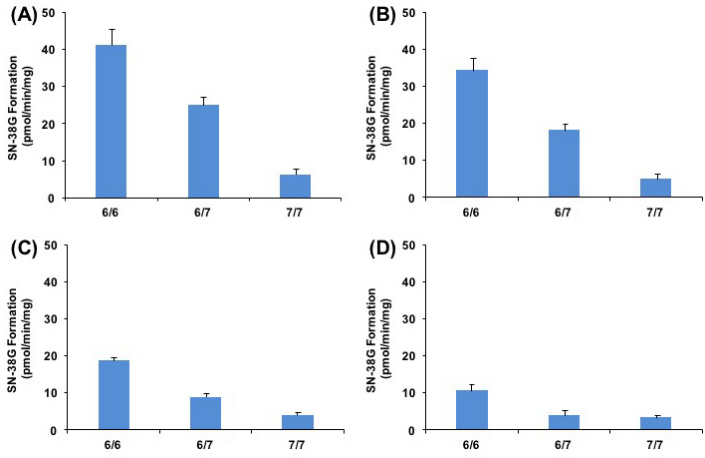
Relationship of UGT1A1*28 genotype with SN-38G formation rates (Vmax) at different concentration of belinostat in presence of 0 **A**., 10 **B**., 100 **C**., or 200 μmol/L **D**., on SN-38 glucuronidation.

Our results also suggested the association of UGT1A1 genetic polymorphism and the inhibitory effect of belinostat on SN-38G formation. As illustrated in Figure 4A, SN-38 glucuronidation activities (Vmax) decreased significantly (*p* < 0.05) in the samples using variant 6/7 and 7/7 genotypes (24.96 pmol/min/mg and 6.23 pmol/min/mg, respectively), as compared to the samples with wild type 6/6 genotypes (41.13 pmol/min/mg). Moreover, co-administration of 10, 100, or 200 μmol/L belinostat and SN-38 led to further reduction in the SN-38G formation rates and this reduction occurred in a dose-dependent manner (Figure 4B-4D). In particular for 200 μmol/L of belinostat (Figure 4D), the rates of SN-38G formation were nearly similar for both variant 6/7 heterozygous and 7/7 homozygous genotypes, at 3.77 pmol/min/mg (decreased by ~ 64% compared to wild type) and 3.30 pmol/min/mg (decreased by ~ 68% compared to wild type), respectively, suggesting that belinostat, at high concentrations, is able to inhibit both heterozygous and homozygous genotypes to a similar extent. This implied that patients with heterozygous genotype may develop severe toxicity similar to that of the homozygous genotype, if a high dose of belinostat is prescribed. Since large populations worldwide are found to have heterozygous UGT1A1*28 genotype and it is thought to be at a lower risk of developing irrinotecan-related toxicity as compared to the homozygous genotype [14, 36], our finding can be clinically relevant as it may prompt extra caution when the combination of irinotecan and belinostat is given to patients with heterozygous genotype. Further investigation is warranted to confirm this finding *in vivo*.

### Effects of vorinostat on SN-38 glucuronidation by UGT1A9

At a therapeutic concentration of vorinostat (2 µmol/L), no significant inhibition of UGT1A9 was observed. CLint of SN-38G increased from 7.48 to 12.12 μL/min/mg protein when incubated without and with vorinostat respectively, but the increase was found to be insignificant (*p* > 0.05).

### Effects of panobinostat on SN-38 glucuronidation by UGT1A isoforms

Panobinostat, at both concentrations (20 and 200 nmol/L), was not found to have any inhibitory effect on all 3 UGT1A isoforms tested. Comparing 20 nmol/L and 200 nmol/L panobinostat to the incubations without panobinostat, the CLint increases were all insignificant (*p* > 0.05).

## DISCUSSION

To our knowledge, this is the first report describing glucuronidation-mediated drug interaction of irinotecan with hydroxamate HDACis. In order to determine the concentration of SN38 and SN38G, a sensitive and robust LC-MS/MS method has been successfully developed and validated according to the FDA guidelines [33]. Excellent linearity was achieved with good precisions and accuracies for SN38 and SN38G. We have demonstrated that belinostat inhibits UGT1A1 non-competitively in a dose-dependent manner by decreasing the glucuronidation of SN-38, the active metabolite of irinotecan. However, this was not the case for vorinostat and panobinostat. This is consistent with other studies, which showed that SN-38 and belinostat are primarily deactivated by UGT1A1 [15, 28]. UGT1A1 has also been reported to have more than 1 binding site, and there are several known non-competitive inhibitors of UGTs enzymes, such as phenylbutazone and quinine [34]. However, the molecular basis of belinostat's non-competitive inhibition is unknown.

There has been interest in developing combination therapy with irinotecan and HDACis like vorinostat [35]. Notably, our results suggested that taking belinostat orally (simulated by its concentration at 10 μmol/L) with irinotecan would not have significant interactions, whereas using a high-dose intravenous belinostat infusion (simulated by its concentration at 200 μmol/L) with irinotecan could pose severe DDI problems. The data suggested that both drugs should not be simultaneously administered together via intravenous infusion. If this combination is needed, human interaction studies should be performed to establish the safety of doses administered. Furthermore, our novel findings can be useful for formulation scientists as they may design combination of anti-cancer products in the future to combat drug-resistant cancers. Our results suggest that irinotecan and belinostat should not be combined and formulated together for use due to their potential DDI, which may cause SN-38 driven toxicity. This finding would be of importance, especially since belinostat was FDA approved in 2014 [31].

Using recombinant UGT1A9, we observed that SN-38G formation was not reduced by vorinostat at its therapeutic concentration of 2 μmol/L, when given orally [32]. Clinically, our results suggested that there should be no drug interaction problem when administering oral vorinostat with intravenous irinotecan. In the future, formulation scientists could combine these 2 drugs in a formulation if it is shown that they have increased efficacy in treating cancer.

Currently, there have not been any studies demonstrating the UGT isoforms that are responsible for the glucuronidation of panobinostat. As such, we decided to study the isoforms (UGT1A1, UGT1A6 and UGT1A7) involved in SN-38 glucuronidation and investigate whether panobinostat inhibits any of these isoforms. In our present work, the results obtained suggested that there is no glucuronidation-mediated DDI between panobinostat and irinotecan via UGT1A1, UGT1A6 and UGT1A7. Hence, there should be no drug interaction problems when administering intravenous irinotecan with oral or intravenous panobinostat. Therefore, irinotecan and panobinostat can be used safely as a combination therapy in a clinical setting, and formulation scientists can formulate such combination products in the future.

Most importantly, it was stated and shown earlier in our findings that genetic polymorphic expression of UGT1A1 can cause inter-individual variability in irinotecan pharmacokinetics and pharmacodynamics [3]. Critically, the DDI between irinotecan and belinostat was found to be worsened to the same extent for both the UGT1A1*28 heterozygous and homozygous genotypes, highlighting the potential clinical significance as a larger proportion of patients with heterozygous genotype may have the same risk of developing severe toxicity as those of the homozygous genotype. It is noteworthy that the Caucasian population is particularly at risk, as 11.5% are UGT1A1*28 homozygotes and 51.4% are heterozygotes, increasing the total population at risk of severe toxicity to 66.9% [36]. Also in Asians, the Indian population is most at risk, as 1.7% are UGT1A1*28 homozygotes and 25% are heterozygotes, bringing more than a quarter of the population (26.7%) to a risk of severe toxicity [37].

Currently, individualised treatment of irinotecan can be tailored for patients who have been genotyped with the UGT1A1*28 allele, which predisposes them to higher risks of neutropenia [36]. However, it has also been shown that UGT1A1*28 is associated with reduced belinostat glucuronidation, which suggests that patients with this genotype may potentially have higher exposure to active belinostat resulting from impaired clearance of belinostat [17, 18, 28]. This has serious implications, as a higher concentration of belinostat due to the UGT1A1 mutation will result in a much stronger inhibition of SN-38 glucuronidation, thereby causing severe toxicity accordingly. Internationally, this polymorphism is also more common in Caucasians and Africans than Asians, with an allelic frequency of about 3-fold or more in Caucasian and African counterparts [38]. Thus, this suggests a greater risk of severe toxicity if combination therapy containing irinotecan and belinostat is given to patients harbouring the commonest genetic variant of UGT1A1.

Therefore, irinotecan and belinostat should be administered together with caution, and our results need careful evaluation in human interaction studies. As the results of our study could be clinical significant due to the high frequency of genetic variants of UGT1A1, we strongly believe that further investigations into the potential inhibition of other drugs including new substrates and inhibitors of UGT1A1 on irinotecan metabolism are warranted due to their severe adverse effects in combination therapies.

## MATERIALS AND METHODS

### Chemicals and reagents

SN-38, SN-38G and their internal standards, SN-38-d_3_ and SN-38G-d_3_ respectively, were obtained from Toronto Research Chemicals (Toronto, ON, Canada). Belinostat, vorinostat and panobinostat, were purchased from Selleck Chemicals (Houston, TX, USA). Human recombinant UDP-glucuronosyltransferases (UGT1A1, UGT1A6, UGT1A7, UGT1A9), UGT control, UGT reaction mix - solutions A and B, HLMs (50 donor pool, UGT1A1*1*28 and UGT1A1*28*28 allelic variants) were purchased from BD Gentest (San Jose, CA, USA). All other reagents were of HPLC grade or of the highest grade commercially available.

### Liquid chromatography-tandem mass spectrometry analysis of SN-38 and SN-38G

The protein precipitation with acetonitrile was adopted for sample preparation for determination of SN-38 and SN-38G, similar toa previously described method in which methanol was used as a a protein precipitating agent [39]. Ten microliter aliquots were injected into a high-performance liquid chromatograph assay system (Agilent 1100, Germany) with tandem mass spectrometric detection (API 4000 triple-quadrupole mass spectrometer; AB Sciex, Concord, Canada). Baseline separation of the analytes was achieved on the Alltima C18 (150 mm x 2.1 mm, 5 μm) column. The mobile phase A and B consisted of 100% acetonitrile and 0.1% formic acid, respectively. Gradient elution was done for 5 minutes at the flow rate of 0.4 mL/min using the following stepwise gradient: 0 to 0.1 minutes, 84% A and 16% B; 0.1 to 1.6 minutes, 16% A and 64% B; and 1.6 to 5 minutes, back to 84% A and 16% B.

### Incubations of SN-38 with belinostat

The incubation mixture (50 µL) consisted of SN-38 (80 - 16000 nmol/L), belinostat at different concentrations (0, 10, 100 and 200 µmol/L), MgCl_2_ (8 mmol/L), Tris-HCl buffer at pH 7.4 (50 mmol/L), alamethicin in dH_2_O (0.025 mg/mL) and UDPGA (5 mmol/L) in Eppendorf tubes. After pre-incubation, the reaction was started with the addition of UGT1A1 or pooled HLMs (0.5 mg/mL). Reaction mixtures were incubated at 37°C for 30 minutes. To ensure clinical relevance, the C_max_ of SN-38 in clinical trials falls within the range being studied [40]. Also, belinostat at 10 µmol/L, 100 µmol/L and 200 µmol/L were chosen as they represented the C_max_ of oral, low-dose and high-dose intravenous administration respectively [41–43]. Appropriate control reactions were also conducted with UGT control as the microsomal protein in a similar fashion.

### Incubations of SN-38 with vorinostat

SN-38 was incubated in the presence of vorinostat (0 and 2 µmol/L) and UGT1A9 (0.5 mg/mL). Vorinostat 2 µmol/L was chosen as it represented the C_max_ following oral administration. UGT1A9 was chosen since vorinostat and SN-38 share this similar minor metabolic pathway [29].

### Incubations of SN-38 with panobinostat

SN-38 was incubated in the presence of panobinostat (0, 20 and 200 nmol/L) and various enzymes in the UGT1A family (UGT1A1, UGT1A6, UGT1A7 at 0.5 mg/mL). Panobinostat at 20 nmol/L and 200 nmol/L were chosen as they represented the C_max_ following oral and intravenous administration respectively [44]. UGT1A1, UGT1A6 and UGT1A7 were chosen since these UGTs could be responsible for glucuronidating panobinostat [45].

### Data analysis

All experiments were carried out in triplicate. SN-38G formation velocity (ν) was calculated as Cm, 30 min/incubation time/microsomal protein concentration, where Cm, 30 min was the metabolite concentration after 30 min incubation. Plots of substrate concentration, S (X axis) versus ν (Y axis) were then constructed. K_m_ and V_max_ were calculated by the Michaelis-Menten Equation 1 below using GraphPad software v5.01 (GraphPad Software, Inc., San Diego, CA, USA). The kinetic parameter V_max_/K_m_, which is equivalent to intrinsic clearance (CL_int_) (Equation 2), was derived from the slope of the plot of the rate of product formation *versus* substrate concentration. Comparisons of CL_int_ between two groups were performed by a paired *t*-test. Data were considered statistically significant when *p* < 0.05.

ν = [S] x V_max_/([S] + K_m_) Equation 1

CL_int_ = V_max_/K_m_ Equation 2
